# Epigenetic remodelling in human hepatocellular carcinoma

**DOI:** 10.1186/s13046-022-02297-2

**Published:** 2022-03-24

**Authors:** Maria Rita Braghini, Oriana Lo Re, Ilaria Romito, Maite G. Fernandez-Barrena, Barbara Barbaro, Silvia Pomella, Rossella Rota, Manlio Vinciguerra, Matias A. Avila, Anna Alisi

**Affiliations:** 1 Unit of Molecular Genetics of Complex Phenotypes, Bambino Gesù Children’s Hospital, IRCCS, Viale S. Paolo, 15, 00146 Rome, Italy; 2 Department of Translational Stem Cell Biology, Research Institute of the Medical University of Varna, Varna, Bulgaria; 3 Hepatology Program, CIMA, University of Navarra, Pamplona, Spain; 4 Center for the Study of Liver and Gastrointestinal Diseases (CIBERehd), Carlos III National Institute of Health, Madrid, Spain; 5 Department of Paediatric Haematology/Oncology and Cellular and Gene Therapy, Bambino Gesù Children’s Hospital, IRCCS, Rome, Italy

**Keywords:** HCC, Epigenetics, Methylation, Non-coding RNAs, Histone modifications, Epidrugs

## Abstract

Hepatocellular carcinoma (HCC) is the most frequent primary liver cancer, being the sixth most commonly diagnosed cancer and the fourth leading cause of cancer-related death. As other heterogeneous solid tumours, HCC results from a unique synergistic combination of genetic alterations mixed with epigenetic modifications.

In HCC the patterns and frequencies of somatic variations change depending on the nearby chromatin. On the other hand, epigenetic alterations often induce genomic instability prone to mutations. Epigenetics refers to heritable states of gene expression without alteration to the DNA sequence itself and, unlike genetic changes, the epigenetic modifications are reversible and affect gene expression more extensively than genetic changes. Thus, studies of epigenetic regulation and the involved molecular machinery are greatly contributing to the understanding of the mechanisms that underline HCC onset and heterogeneity. Moreover, this knowledge may help to identify biomarkers for HCC diagnosis and prognosis, as well as future new targets for more efficacious therapeutic approaches.

In this comprehensive review we will discuss the state-of-the-art knowledge about the epigenetic landscape in hepatocarcinogenesis, including evidence on the diagnostic and prognostic role of non-coding RNAs, modifications occurring at the chromatin level, and their role in the era of precision medicine.

Apart from other better-known risk factors that predispose to the development of HCC, characterization of the epigenetic remodelling that occurs during hepatocarcinogenesis could open the way to the identification of personalized biomarkers. It may also enable a more accurate diagnosis and stratification of patients, and the discovery of new targets for more efficient therapeutic approaches.

## Background

Hepatocellular carcinoma (HCC) is currently one of the most leading causes of death worldwide, being the sixth most commonly diagnosed cancer and the fourth leading cause of cancer-related death, with approximately 854.000 new cases in 2015 [1, 2].

HCC corresponds to 75–85% of primary liver cancers [1]. The bad incidence of HCC is attributed to its highly complex heterogeneity and to the plurality of the aetiologies. Among the well-defined risk factors for HCC development there are cirrhosis, hepatitis B virus (HBV) and hepatitis C virus (HCV) infection, alcohol abuse and non-alcoholic steatohepatitis (NASH). Moreover, obesity, diabetes, insulin resistance, smoking, and dietary exposure to aflatoxins are relevant cofactors that contribute to the onset of HCC [3]. HCC commonly arises in patients with underlying liver disease, mostly as a result of HBV or HCV infection or alcohol abuse. However, the increase in non-alcoholic fatty liver disease (NAFLD) will soon become the major leading cause of liver cancer in Western countries [4]. Currently, more and more HCC cases, including the paediatric ones, occur in the absence of cirrhosis mainly in the context of NAFLD/NASH or chronic HBV infection [5–7].

Apart imaging examinations and biopsies, the biomarker commonly used for the screening of HCC is the circulating α-fetoprotein (AFP) that, however, has an unsatisfied diagnostic efficiency in the screening of early-stage HCC for its elevations in patients with liver benign diseases, such as cirrhosis and chronic hepatitis [8]. Therefore, the research of new and more accurate biomarkers for HCC becomes urgent, especially for an early diagnosis and to monitor the outcome of the disease after treatment [9, 10].

The HCC is highly refractory to treatments and continues to have a high recurrence rate, but the scenario of available therapies for HCC has improved during the last five years [11]. Indeed, the recent technological progresses for conventional therapeutic options, as well as the development of new promising targeted systemic therapy and immune checkpoint inhibitors have increased the life-expectancy of patients with advanced HCC [11, 12]. Despite this step forward, it has been reported an high incidence of recurrent de novo HCC tumours, which progress into incurable, advanced-stage condition for which the available medical therapies could have only marginal survival benefit and are not cost-effective [13].

The understanding why some HCCs are refractory and recur after the current treatments remains as open question. However, HCC heterogeneity, which depends on genetics, epigenetics, transcriptomics, proteomics, and metabolomics, could be the answer to this question [14–16]. Moreover, HCC consists of malignant cells embedded in a tumour microenvironment that is critical for either inhibiting or enhancing the efficiency of all the steps of carcinogenesis. Indeed, the close interaction between the malignant cells and the tumour microenvironment paves the way to epigenetic processes able to modify chromatin allowing DNA accessibility, thus modifying the gene expression. Therefore, the HCC heterogeneity could be the result of a unique synergistic combination of genetics mixed with epigenetics [17]. Epigenetics includes three main heritable regulatory systems that determine chromatin remodelling and gene transcription regulation: non-coding RNAs, DNA methylation and chromatin remodelling, and the functional crosstalk among these epigenetic processes determines cell phenotype [18].

All these lines of evidences highlight that studies of the epigenetics may give a great contribute to the comprehension of the mechanisms that underline HCC onset and progression, thus revealing new targets for future more efficacious therapeutic approaches or new biomarkers for HCC diagnosis.

**Fig. 1:**
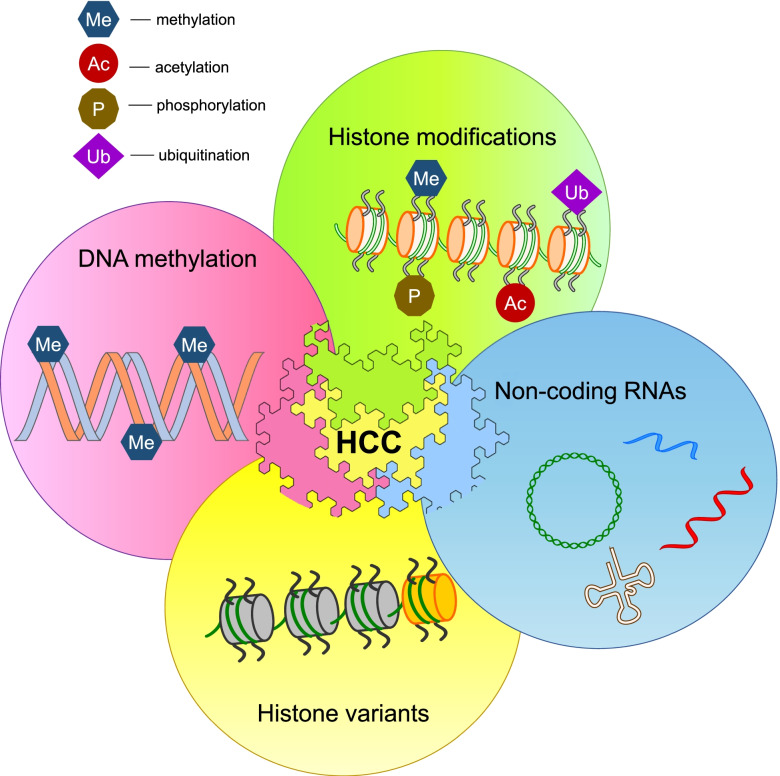
The regulatory systems involved in the epigenetic landscape of human HCC. The epigenetic marks in HCC include histone tails modifications (acetylation, methylation, phosphorylation, etc.), non-coding RNAs, the presence of abnormal histone variants, and DNA promoter hypo- or hyper-methylation. The post-translational modifications of histone tails can modulate chromatin organization and alter the control of gene expression. Non-coding RNAs have emerged as regulators of chromatin structure. Non-canonical histone variants can affect chromatin remodelling and histone post-translational modifications. Alterations in the methylation of gene promoter regions cause altered gene expression

In this comprehensive review we discuss the epigenetic landscape investigated in the last decade in human HCC (Fig. 1).

### Non-coding RNAs

Non-coding RNAs (ncRNAs) comprise multiple classes of RNAs that are not translated into proteins. They are a heterogeneous group of molecules that have emerged as regulators of chromatin structure and function with crucial roles in both physiological and pathological conditions [19]. Altered expression of ncRNAs has been reported in many disease settings, as well as in many types of cancer, including HCC [20]. They are classified into two major group types based on length. The first group is represented by small/short ncRNAs (sncRNAs) with a length < 200 nucleotides, mainly represented by microRNAs (miRNAs), piwi-interacting RNAs (piRNAs), endogenous short interfering RNAs (endo-siRNAs) and small nucleolar RNAs (snoRNAs). The second group includes ncRNAs with a length > 200 nucleotides known as long ncRNA (lncRNAs) [21]. Relatively recent is the discovery of a further class of ncRNAs called circular RNAs (circRNAs) [22].

#### Hepatic and circulating miRNAs and HCC

The most widely studied ncRNAs are miRNAs, approximately 22 nucleotides-long single-stranded RNAs, which mostly function as negative gene regulators at post-transcriptional level. Defects in the complex process of miRNA biogenesis and exportation may lead to a global deregulation of miRNA expression, as well as that observed in human HCC [23, 24]. miRNAs can regulate gene expression by inducing cleavage/translational repression of their complementary to the 3′-untranslated region (3ʹUTR) of mRNA targets [25]. Multiple profiling studies have highlighted that miRNA expression is substantially different between human tumour and non-tumour livers. miRNAs that normally exert tumour-suppressive effects by repressing oncogenes are recurrently down-regulated in HCC, such as miR-15a/16–1, miR-122, miR-139 miR-192, miR-199a/b-3p and miR-275 [26–34]. On the other hand, the miRNAs known as ‘oncomiRs’ promote oncogenesis by negatively regulating important tumour-suppressive protein-coding genes, and they are aberrantly increased in HCC. Among the most representative oncomiRs there are miR-21, miR-103a, miR-221, miR-222 [28–30, 35]. However, in HCC, miRNAs may target also several cellular signalling hubs involved in innate and adaptive immune cell responses and tumour microenvironment [36–39]. Interestingly, in patient-based studies several hepatic miRNAs have been associated to HCC outcome, suggesting their potential use as biomarkers. In particular, the up-regulation of DLK1-DIO3 miRNA cluster and miR-10b, and the down-regulation of miR-199a/b and miR-122 have been correlated with poor prognosis; while the up-regulation of miR-135a, and down-regulation of miR-99a have been correlated with poor survival (see the review by Gupta and Akhtar) [36].

During the last decade also several circulating miRNAs, released from cancerous tissues as free miRNAs or contained within the exosomes, have been identified and tested for their efficiency as biomarkers in HCC diagnosis and prognosis (Table 1) [40–50].

**Table 1:** List of the main sets of circulating miRNAs described as modulated in HCC

miRNAs sets	Pattern of regulation in HCC patients compared to controls	Reference
***miR-122***	Up	[40]
***miR-21***	Up	
***miR-223***	Down	
***miR-885-5p***	Up	[41]
***miR-574-3p***	Up	
***miR-224***	Up	
***miR-215***	Up	
***miR-146a***	Up	
***miR-15b***	Up	[42]
***miR-130b***	Up	
***miR-206***	Up	[43]
***miR-141-3p***	Up	
***miR-433-3p***	Up	
***miR-1228-5p***	Up	
***miR-199a-5p***	Down	
***miR-122-5p***	Down	
***miR-192-5p***	Down	
***miR-26a-5p***	Down	
***miR-29a***	Up	[44]
***miR-29c***	Up	
***miR-133a***	Up	
***miR-143***	Up	
***miR-145***	Up	
***miR-192***	Up	
***miR-505***	Up	
***miR-939***	Up	[45]
***miR-595***	Up	
***miR-519d***	Up	
***miR-494***	Up	
***miR-224***	Up	[46, 47]
***miR-92-3p***	Up	[48]
***miR-107***	Up	
***miR-3126-5p***	Down	
***miR-1972***	Up	[49]
***miR-193a-5p***	Up	
***miR-214-3p***	Up	
***miR-365a-3p***	Up	
***miR-1246***	Up	[50]
***miR-122***	Up	[51]
***miR-155***	Down	
***miR-885-5p***	Up	
***miR-221-3p***	Down	[52]
***miR-223-3p***	Down	
***miR-26a-5p***	Down	
***miR-30c-5p***	Down	
***miR-365a-3p***	Up	
***miR-423-3p***	Up	

Some studies highlighted that altered circulating miRNAs may be biomarkers for liver injury but not specific for HCC [40, 41, 45, 49], and revealed that the serum levels of miR-21, miR-122 and miR-223 were found elevated in HCC but also in chronic hepatitis B patients. However, the large part of the literature identified specific circulating miRNA signatures in HCC compared with healthy or cirrhotic livers in monocentric or multicentric studies [42–44, 46–48, 50, 51, 53]. Interestingly, a systematic review highlighted that plasma levels of miR-224 in HCC patients were higher than in healthy volunteers, and were significantly correlated with larger tumour size and recurrence. The authors also highlighted that the plasma miR-224 levels could accurately identify small tumours less than 18 mm in diameter [46]. Lin et al. confirmed the high serum level of miR-224 in early-stage HCC respect to cirrhosis, chronic hepatitis B and C [47]. In addition, Yamamoto et al. [51], performed the analysis of circulating miRNAs by microarrays in serum samples from 345 patients with HCC, 46 patients with chronic hepatitis, 93 patients with liver cirrhosis, and 1.033 healthy individuals. The authors developed a detection model of HCC, including early stage, based on a set of 52 selected miRNAs that had altered expression according to disease progression status. More recently, Wong et al. [53] showed a six miRNA signature, including miR-221-3p, miR-223-3p, miR-26a-5p, miR-30c-5p, miR-365a-3p, and miR-423-3p, which were significantly differentiated in HCC compared to healthy individuals.

Finally, several authors have demonstrated the value of circulating miRNAs also as biomarkers of the follow-up after different types of treatments [52, 54, 55].

What is evident from the survey by Shen et al. [56] is that miRNA signatures in HCC are dependent on etiological factors and ethnic groups, thus explaining why miRNAs are different across the cohorts studied, and their application as biomarkers requires further investigation.

#### piRNAs and HCC

piRNAs include a large family of small (26–31 nucleotides), single stranded ncRNAs that interact with the PIWI subfamily of argonaute proteins to form functional RNA-protein complexes that may play a role in cancer [57, 58].

However, only a few studies have clarified the role of piRNAs in HCC (Table 2). Law et al. [59] found the up-regulation of piR-Hep1 in 46.6% of hepatic tumours when compared with the corresponding adjacent tissues. Rizzo et al. [60] investigated piRNAs expression by RNA-Seq in cirrhotic and HCC samples, and stringent analyses revealed a set of 125 piRNAs that characterize HCC from matched cirrhotic nodules, and correlating also to microvascular invasion. Moreover, the same authors showed a specific expression of 24 piRNAs in dysplastic nodules respect to cirrhotic liver and/or progressed HCC. The latest study by Koduru et al. [32] recognized 128 piRNAs in HCC (83 up-regulated and 45 down-regulated). The top five up-regulated piRNAs include piR-32,299, piR-23,670, piR-24,684, piR-28,488, and piR-7239; while, the top five down-regulated were piR-952, piR-820, piR-28,525, piR-5938, and piR-5937. The most relevant piRNAs emerged by the above mentioned studies are listed in Table 2.

**Table 2:** List of the main piRNAs described as modulated in HCC

piRNAs	Pattern of regulation in HCC patients compared to controls	Reference
***piR-Hep1***	Up	[59]
***hsa-piR-001078***	Up	[60]
***hsa-piR-001207***	Up	
***hsa-piR-001346***	Up	
***hsa-piR-017061***	Up	
***hsa-piR-017295***	Up	
***hsa-piR-019420***	Up	
***hsa-piR-020450***	Up	
***hsa-piR-001170***	Down	
***hsa-piR-016975***	Down	
***hsa-piR-017724***	Down	
***hsa-piR-019951***	Down	
***hsa-piR-020828***	Down	
***hsa-piR-020829***	Down	
***piR-LLi-7***	Down	
***piR-LLi-54***	Down	
***piR-LLi-1977***	Down	
***piR-LLi-13,491***	Down	
***piR-LLi-24,259***	Down	
***piR-LLi-28,197***	Down	
***piR-LLi-30,517***	Down	
***piR-LLi-30,581***	Down	
***hsa-piR-020498***	Down	
***piR-LLi-30,552***	Down	
***piR-LLi-24,894***	Down	
***hsa-piR-013306***	Down	
***piR-32,299***	Up	[32]
***piR-23,670***	Up	
***piR-24,684***	Up	
***piR-28,488***	Up	
***piR-7239***	Up	
***piR-952***	Down	
***piR-820***	Down	
***piR-28,525***	Down	
***piR-5938***	Down	
***piR-5937***	Down	

However, most of the studies investigate the role of genes involved in piRNAs machinery, such as Piwi and Hiwi [61–63].

#### snoRNAs and HCC

snoRNAs are a highly conserved subclass of sncRNAs of 60–300 bp localized into the nucleolus, where they contribute to maturation of RNA molecules through chemical modifications targeting mainly rRNAs, tRNAs, and sncRNAs [64]. In the recent years, have demonstrated that dysregulated expression of snoRNAs may play a crucial role in HCC development (Table 3).

**Table 3:** List of the main snoRNAs described as modulated in HCC

snoRNAs	Pattern of regulation in HCC patients compared to controls	Reference
***SNORD113–1***	Down	[65]
***SNORD78***	Up	[66]
***ACA11***	Up	[67]
***SNORD113–1***	Down	[68]
***SNORA47***	Up	
***SNORD126***	Up	
***SNORD78***	Up	
***SNORD76***	Up	
***snoU2_19***	Up	[69]
***SNORD76***	Up	[70]
***SNORA24***	Down	[71]
***SNORD72***	Up	[72]

Xu et al. found that over-expression of SNORD113–1 suppressed tumorigenicity through inactivation of MAPK/ERK and TGF-β pathways in vitro and in vivo HCC models [65]. A study by Ma et al. [66] found that SNORD78 was up-regulated in HCC tissues respect to adjacent non-neoplastic tissues. Furthermore, their results showed that high expression of SNORD78 was associated with aggressive tumour phenotype and poor outcome of HCC patients. In addition, loss-of-functional analyses indicated that SNORD78 silencing inhibited cell growth, invasion, and migration in vitro, suggesting that SNORD78 was associated with development and progression of HCC.

Wu et al. [67] demonstrated that the snoRNA ACA11 was higher expressed in HCC tissues than in adjacent non-tumour tissues, correlating also with shorter overall survival and higher recurrence rates. This study provides a novel evidence of a potential diagnostic role of ACA11 in HCC.

To date, new studies are discovering new snoRNAs differential expression signature in HCC tissues. Indeed, in tumours compared with non-tumour tissues, it has been found a down-regulation of SNORD113–1 associated with worse survival, and an up-regulation of SNORA47, SNORD126, SNORD78 and SNORD76 linked to the aggressive phenotype and poor prognosis of HCC [68].

In addition, more recently it has been reported that up-regulated snoU2_19 was associated with HCC progression and aggressive phenotypes [69].

Furthermore, the biological role of SNORD76 has been clarified in HCC development, demonstrating for the first time that SNORD76 can be used as a prognostic biomarker. Indeed, the authors found a significant up-regulation of SNORD76 in 66 HCC specimens compared to non-tumour tissues that associated with poor survival [70].

Recently, McMahon et al. [71] provided evidence that one snoRNA plays a direct role in specific steps of cancer development. The study revealed that SNORA24 levels, which mediates two distinct pseudouridine modifications in the small 40S subunit of the ribosome, were decreased in HCC and is implicated in a tumour suppressor program that blocks cellular transformation. Moreover,  human and mouse data support a role for SNORA24 dysfunction in tumour initiation and in maintenance of RAS-driven cancers. Interestingly, in the context of HCC, SNORA24 appears to be unnecessary for protein synthesis, but it is involved in more selective functions, for example towards controlling translation of specific mRNAs.

In a recent study Mao et al. [72] revealed that the knockdown of lncRNA-LALR1 up-regulated the snoRNA SNORD72 via binding with SNORD72, and stabilized ID2 mRNA. SNORD72 was over-expressed in HCC tissues and enhanced cells proliferation, colony formation and invasion. Interestingly, a recent comprehensive analysis provided a classification of the snoRNAs correlated to this tumour [73].

#### lncRNAs and HCC

lncRNAs, with a length > 200 nucleotides, exert different biological functions depending on their localization into specific cellular compartments [74]. Nuclear lncRNAs can act as a guide for chromatin-modifying-complexes or transcription factors; cytoplasmatic lncRNAs often function as regulators of protein levels, either by directly controlling mRNA stability or by acting as the so-called competing endogenous RNAs, that sequester miRNAs.

A large amount of studies demonstrated that lncRNAs may play an important role in the development and progression of HCC, thus they could be promising targets for therapy, but also promising biomarkers with high accuracy and efficiency for diagnosis and prognosis [75–77]. Since the role of hepatic lncRNAs has been extensively investigated and deserves a separate discussion, here we focused only on the circulating lncRNAs that differed in patients with HCC respect to healthy subjects (Table 4) [55, 76–86].

**Table 4:** Circulating lncRNAs altered in HCC

lncRNAs	Pattern of regulation in HCC patients compared to controls	Reference
***RP11–160H22.5***	Up	[78]
***LOC149086***	Up	
***XLOC_014172***	Up	
***HULC***	Up	[79]
***LINC00152***	Up	
***CTBP***	Up	[80]
***RP11–160H22.5***	Up	[81]
***XLOC_014172***	Up	
***LINC00152***	Up	
***LINC00161***	Up	[82]
***TSIX***	Up	[83]
***GAS5***	Down	[84]

Among the circulating lncRNAs, RP11–160H22.5, LOC149086, and XLOC_014172 were found up-regulated in HCC [78]. Li et al. [79] reported that the plasma expression levels of HULC and LINC00152 may discriminate between HCC and controls. Also the lncRNA CTBP was significantly up-regulated in serum, thus it can serve as a potential biomarker for HCC diagnosis [80]. Again, LINC00152, RP11-160H22.5, and XLOC014172 were found up-regulated in the plasma of patients with HCC by Yuan et al. [81]. Sun et al. [82] demonstrated that LINC00161, up-regulated in the serum of patients with HCC, may be a novel biomarker for this type of tumour. Moreover, Habieb et al. [83] found in HCC patients increased serum levels of TSIX, a lncRNA antisense for X-inactive-specific transcript. The plasma levels of GAS5 were significantly lower in HCC patients compared to healthy subjects [84]. Furthermore, this lncRNA, as well as the exosomal H19, could be counted as a biomarker for response to treatment with sorafenib [55, 85]. Finally, recently the circulating levels of some lncRNAs have been correlated with metastasis and poor prognosis in HCC [86].

#### CircRNAs and HCC

circRNAs are particular non-coding RNAs formed by back-splicing events, a “head-to-tail” attachment, leading to covalently closed loop structures, thus without the presence of 5′ and 3′ ends [87, 88]. They are highly stable and can be detected in cell-free compartments such as exosomes and plasma, suggesting circRNAs as biomarkers for cancer diagnosis. High-throughput RNA sequencing technology has highlighted a large number of circRNAs, which are endogenous, abundant and stable in mammalian cells, and that may be involved in many diseases including cancer [89, 90]. Recently, many reports suggested that circRNAs had special effects on the occurrence and development of liver disease [91]. Many circRNAs are differentially expressed in HCC tissues, indicating a potential function in their process [92].

Among the most up-regulated circRNAs in HCC tissues there are CDR1as/ciRS-7 (a circular RNA sponge for antisense of microRNAs-7 or CDR1) and hsa_circ_0005075, while among the most down-regulated are included circZKSCAN1, hsa_circ_0001649, hsa_circ_0005986 and hsa_circ_0004018 [93]. CDR1 inhibits the expression of microRNAs-7, which can stop the growth of cancer cells and promote apoptosis, thereby increasing the expression of its target genes. As a result, it can promote the initiation and development of cancer [94]. The up-regulation of CDR1 in HCC tissues was observed in patients whose AFP level ≥ 400 ng/ml, suggesting CDR1 as a valuable biomarker for HCC treatment [95].

The up-regulation of hsa_circ_0005075 and the down-regulation of hsa- circ_0001649 expression were correlated with HCC tumour size [96]. Circ-0001649 served as a competing endogenous RNA (ceRNA) to sponge miR-127-5p, miR-612 and miR-4688, thus activating SHPRH, a gene ubiquitously expressed, containing motifs of some DNA repair proteins, transcriptional factors, and helicases [97]. The absence of circZKSCAN1 endowed several malignant properties, including cancer stemness, and tightly correlated with worse overall and recurrence-free survival rate in HCC [98].

More recently, it has been described a cluster of 70 circRNAs specifically expressed in HCC specimens, which included 26 circRNAs up-regulated and 44 circRNAs down-regulated. The most five up-regulated circRNAs were: circR-0021905, circR-0016456, circR-0015637, circR-0087119, and circR-0014848. On the contrary, the most five circRNAs down-regulated were: circR-0035409, circR-0035407, circR 0076872, circR-0092360, and circR-0069970 [32]. Noteworthy, two very recent studies highlighted the role of circ-LRIG3 and circSOD2 as interactor/regulators of other epigenetic circuits involved in the development and progression of HCC [99, 100].

### Chromatin remodelling

The chromatin organization as a dynamic structure consisting of DNA and histone proteins was described for the first time by Kornberg and Thomas [101]. The basic units of chromatin are represented by nucleosomes, histone octamer cores around which DNA is wrapped, and consisting of two copies each of histones H2A, H2B, H3, and H4 [102]. The formation of specific nucleosome arrays along the genome confers to local regions different structural and functional chromatin states, such as euchromatin (transcriptionally active open form) or heterochromatin (transcriptionally silent closed form) [103]. These chromatin conformations are essentially controlled by chemical modifications on DNA (i.e. DNA methylation) and histones that partly define the epigenome. N-terminal tails of histone proteins, protruding out of the nucleosome, are subjected to a variety of post-translational modifications (PTMs), for example methylation and acetylation of arginine and lysine residues, phosphorylation of serine and threonine residues, ubiquitination and sumoylation [104]. All PTMs are catalysed by different families of enzymes (e.g. acetyltransferases, deacetylases, methyl-transferases and demethylases, etc.) that, according to their effect on gene transcription, are classified as activating or repressing enzymes [105].

#### DNA methylation and HCC

Methylation is a heritable enzyme-mediated chemical modification that occurs on the 5 position of the Cytosine ring and is mediated by DNA methyltransferases (DNMTs) [106]. DNA methylation mainly affects the Cytosine base (C) when is followed by a Guanine (G), the so-called CpG sites. In human DNA, 70–80% of CpG sites are methylated; however, methylation is mostly found in areas where CpG density is low, or at repetitive DNA sites. On the contrary, CpG islands (CpGIs) are completely unmethylated in healthy individuals, and represent approximately 1 to 2% of human genome [107]. In general, methylation in the gene promoter region causes transcriptional repression, while methylation in the gene body promotes gene expression, as reported in most cancers including HCC [108]. Among the DNMTs, DNMT1 is essential for the maintenance of methylation pattern in the genome, by regulating gene expression but also other epigenetic regulators (e.g. miRNAs) in HCC [109–111].

Defects in DNA methylation and its machinery are closely associated with cancer, including HCC [112]. Three types of alterations in DNA methylation occur in cancer: hypermethylation of the CpGIs in the promoter regions of tumour suppressor genes, aberrant expression of DNMTs, and global hypomethylation of genes and repetitive sequences leading to genomic instability and oncogene activation [113].

In the last decade, numerous studies highlighted different methylome patterns in HCC, compared to normal tissues through genome-wide DNA methylation profiling. The majority of these studies in HCC focused on finding significantly hypermethylated genes, and among these CDKN2A, RASSF1, APC and SMAD6 emerged [16, 107, 108, 113–125]; while fewer studies described hypomethylated genes, such as IGF2, CCL20, NQO1 [107, 118, 120, 121]. The main hyper- or hypomethylated genes in HCC are listed in Table 5.

**Table 5:** The most relevant hyper- and hypomethylated genes in HCC

Genes	DNA Methylation pattern in HCC	Reference
***HHIP, PTGR1, TMEM106A, MT1M, MT1E, CPS1, CDKN2A***	Hyper	[16]
***CDKN2A, MLH1, FZD7, SOCS1, APC, RASSF1***	Hyper	[107]
***HIST1H2AJ, SPDYA***	Hyper	[108]
***HRNBP3***	Hypo	
***CDKN2A***	Hyper	[113]
***FZD7, CDKN2A, APC, RASSF1***	Hyper	[114]
***SOCS1, RASSF1***	Hyper	[115]
***CHFR, MGMT, RASSF1, GSTP1***	Hyper	[116]
***CDKN2A, BMP4, NFATC1, GSTP1***	Hyper	[117]
***APC, RASSF1***	Hyper	[118]
***IGF2***	Hypo	
***SOCS1, APC, GSTP1***	Hyper	[119]
***SMAD6, IFITM1, LRRC4, CHST4, TBX15***	Hyper	[120]
***CCL20, NQO1***	Hypo	
***p16, CDH1, p15, RUNX3, SFRP1, PRDM2, p14, RARβ, DAPK1, SOCS1, APC, RASSF1, MGMT, GSTP1***	Hyper	[121]
***OCIAD2***	Hyper	[122]
***RASSF1A***	Hyper	[123]
***CDCA1, CDCA3, CDCA4, CDCA5, CDCA6, CDCA8***	Hypo	[124]
***CDCA2, CDCA7***	Hyper	
***MTFR2***	Hyper	[125]

Nowadays, the new generation of sequencing platforms allows the preparation of genomic maps of DNA methylation, permitting the identification of DNA methylation patterns highly rearranged in HCC. Indeed, Meunier et al. identified various processes remodelling HCC methylomes and revealed the epigenetic and transcriptional impact of different alterations [126].

#### Histone modifications and their specific impact on HCC

**Fig. 2:**
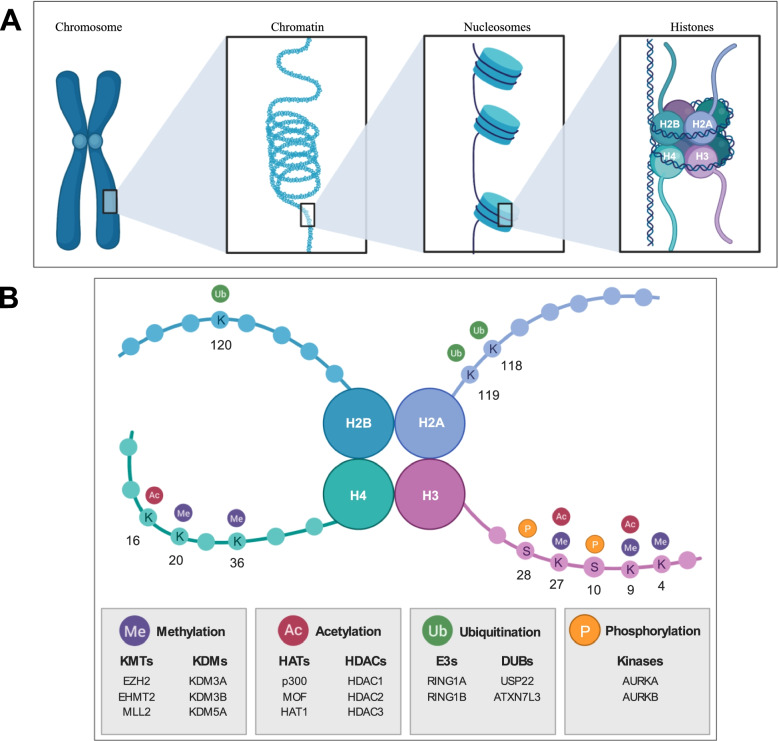
Histone modifications in HCC. **A** Schematic representation of DNA-histone protein complexes. Human chromosomal DNA is made by looped chromatin fibers, tightly coiled around nucleosomes. Each nucleosome is composed of DNA and eight histone proteins (H2A, H2B, H3 and H4 dimers). **B** Schematic representation of the nucleosome. The main PTMs (methylation, acetylation, ubiquitination and phosphorylation) and their localization on the tails of the 4 main histones are reported. Enzymes involved in the mentioned modifications are listed in gray boxes. KMTs: Lysine Methyltransferases; KDMs: Lysine Demethylases; HATs: Histone Acetyltransferases; HDACs: Histone Deacetylases; E3s: E3 Ubiquitin Ligases; DUBs: Deubiquitinating enzymes. Created with BioRender.com (https://biorender.com7), accessed 10 Feb. 2022

As said, DNA is organised into chromatin, composed by nucleosomes wrapped around a histone octamer (Fig. 2). The tails of the histones can be modified and these PTMs can modulate chromatin compaction and control the recruitment of remodelling complexes and transcription factors. This is achieved through the recognition of histone PTMs by proteins harbouring specialized domains that recognize specific covalent modifications, the so-called epigenetic readers. Alterations in histone modifying enzymes cause the deregulation of histone marks, and consequently the control of chromatin-associated processes, which further contribute to malignant cancer hallmarks [18].

##### Histone methylation

Methylation of lysines on histones is controlled by various methyltransferases and demethylases and is an important component of epigenetic histone modifications. Abnormal regulation of such enzymes is frequently found in HCC, thus comporting an altered regulation of gene expression. In fact, a recent study identified 11 methyltransferases and demethylases, such as EZH2, EHMT2, SETDB1 and SETD2 associated with the clinical characteristics of tissues from HCC patients respect to normal liver tissues, confirming the fundamental role of histone methylation regulation [127].

The major sites of histones methylation are lysine 4, 9 and 27 of histone H3. Generally, histone 3 lysine 4 methylation (H3K4me) is associated with gene activation, while histone 3 lysine 9 methylation (H3K9me) and histone 3 lysine 27 methylation (H3K27me) result in gene repression [128].

H3K4 can be mono-, di- or tri-methylated by different enzymes. SET7 is the only methyltransferase which can specifically monomethylate H3K4 and plays critical roles in various diseases, including breast cancer, HCV, atherosclerotic vascular disease, diabetes, prostate cancer and HCC [129].

H3K9 methylation is another epigenetic marker commonly associated with the pathogenesis of HCC, where the global levels of H3K9me2 and H3K9me3 are commonly found higher in cancer tissues than in normal liver tissues, and both H3K9me2/3 are positively correlated with the degree of tumour differentiation and with poor prognosis [130].

Regarding H3K27 methylation, this modification plays a crucial role in repressing gene transcription during developmental stage and cellular differentiation, and is mediated by polycomb group proteins, that consequently have a key role in oncogenesis of many types of cancer, including HCC [131]. The polycomb proteins are assembled into polycomb repressive complex 1 (PRC1) and polycomb repressive complex 2 (PRC2) to generate two different histone modifications: monoubiquitination of lysines 118 and 119 in histone H2A (H2AK118ub and H2AK119ub) and H3K27me1/2/3, respectively. The PRC2 complex, responsible for H3K27 methylation, is composed by enhancer of zeste homolog 1 and 2 (EZH1/2), suppressor of zeste 12 (SUZ12), the embryonic ectoderm development (EED) and retinoblastoma-binding protein 4/7 (RBBP4/7) [132]. Among these proteins, EZH2 is the most studied. In line with this, Hung et al. [133] showed an up-regulation of different histone modifications markers in 50 paired tumour tissues respect to adjacent non-cancerous tissues and, among others, EZH2 was associated with tumour stage and survival. EZH2-mediated H3K27me3 represents a major oncogenic chromatin modification, but is not well known how it modulates the signalling pathways. A recent study showed that sorafenib-induced AKT inhibition in HCC resulted in an increase of H3K27me3 levels through a decrease of EZH2 phosphorylation, generating a mechanism of sorafenib-resistance in HCC. This study suggests that a combined treatment of sorafenib plus EZH2 inhibitors may represent a novel therapeutic approach in HCC [134]. We observed that in human HCC the up-regulation of EZH2 was correlated with increased levels of focal adhesion kinase (FAK) and H3K27me3. This study evidenced a new molecular nexus between EZH2 and FAK that may control HCC growth [135, 136], and that has been correlated with poor prognosis in paediatric HCC [137, 138].

Other examples of altered histone H4 lysine methylation in HCC involve lysines 20 and 36. A recent study demonstrated that the development of NASH-related HCC is characterized by a global loss of histone H4 lysine 20 trimethylation (H4K20me3), and global and gene-specific deacetylation of histone H4 lysine 16 (H4K16). As a consequence, altered gene expression resulted in inhibited cell death [139].

##### Histone acetylation

Among the epigenetic changes that regulate gene expression, histone acetylation is one of the most significant and has taken clinical diagnostic significance in various types of cancer, including HCC [140]. Histone acetylation is controlled by two groups of enzymes, histone acetyltransferases (HATs) and histone deacetylases (HDACs), working in an accurate and regulated manner to open the chromatin and transcriptionally activate the expression of determined genes [141].

p300, the HAT most studied, was found to mediate H3K9 acetylation (H3K9ac) after phosphorylation elicited by EGF in HCC cells. This signalling pathway was correlated with high expression of High-mobility group protein A2 (HMGA2), frequently up-regulated in HCC and associated with metastasis and poor survival, and the knockdown of p300 reversed the EGF-induced HMGA2 expression and histone H3K9ac [142]. Another HAT, called MOF, plays critical roles in the transcription activation by acetylating histone H4K16. MOF was found down-regulated in HCC, and this down-regulation correlated with poor overall survival. Moreover, MOF knockdown promoted HCC growth, whilst MOF over-expression reduced it, suggesting that MOF participates to HCC progression [143]. The role of MOF has also been studied in association with the process of vascular invasion in HCC. In fact, H4K16ac was identified as a biomarker of microvascular invasion in HCC, and a study showed that MOF down-regulation caused a decrease of expression in genes and pathways involved in vascular invasion in HCC cells. Furthermore, MOF down-regulation significantly reduced tumour cell intravasation and metastasis [144]. Among the HATs, also the expression of HAT1 was up-regulated in HCC specimens respect to normal liver tissues, and its protein level was increased with tumour stage [145].

Interestingly, the H3K27ac serves in part to counteract PRC2 action, since acetylation precludes methylation by PRC2 at this site. Accordingly, we recently showed in HCC that a combined therapy with a FAK inhibitor plus sorafenib caused a decrease of the global levels of H3K27me3, of the protein levels of HDAC1 and 2, and consequently H3K27ac increased [146].

Because of the fundamental role of histone acetylation, inhibitors targeting HDACs and HATs have been developed and are used in different preclinical and clinical studies for cancer therapy, including HCC, where several evidences suggest that epigenetic therapies could be used for its treatment, and numerous studies employed inhibitors with promising results against tumour growth [147].

##### Histone ubiquitination

As previously mentioned, H2AK118ub and H2AK119ub are modifications caused by PRC1. As for PRC2, the catalytic activity of PRC1 is associated with transcription repression, especially the formation of H2AK119ub [148].

A recent study suggested that Hepatitis C virus infection, which is cause of liver pathologies including HCC, impairs H2AK119ub in the homeobox (HOX) gene promoter by destabilizing RNF2, a PRC1 component, and thus inducing HOX genes expression. These findings reveal a novel mechanism of Hepatitis C virus-related histone modification that may contribute to HCC induction [149].

Another epigenetic marker involved in HCC progression is the Ubiquitin-specific protease 22 (USP22), responsible of the deubiquitination of both histones H2A and H2B. Histone deubiquitination is often linked to transcriptional activation, epigenetic regulation and cancer progression. Tang et al. showed that the expression level of USP22 was significantly higher in HCC than in normal liver tissues, and it was significantly correlated with clinical stage, tumour grade and shortened survival time [150]. Moreover, different studies demonstrated that USP22 levels were responsible for the altered HCC drug-resistant phenotype [151–153]. Furthermore, it has been shown that the H2B deubiquitination by ATXN7L3 protein enhanced SMAD7 expression, which acts to inhibit tumour growth in HCC [154].

##### Histone phosphorylation

Like histone acetylation, histone phosphorylation has been associated with active gene transcription, but it has not been well characterized so far. Aurora kinases are responsible for the phosphorylation of most of serine/threonine residues of the histone H3. Aurora A (centrosome-associated) is frequently over-expressed in HCC patients, correlating with high grade tumours. It has been shown that it is able to reduce radiotherapy-induced apoptosis by activating NF-κB signalling, thereby contributing to HCC radioresistance [155]. Aurora B (chromosomal passenger protein) directly phosphorylates histone H3 at Ser10 (pH3S10) and Ser28 (pH3S28), thus contributing to chromosome instability and mitotic chromosome condensation. Aurora B is considered an independent molecular marker, predicting tumour growth and invasion of HCCs [156]. Liu et al. observed that Aurora A and B tended to be parallelly over-expressed, and their concurrent over-expression associated with the worst prognosis, being an independent predictor for the survival [157]. It has been demonstrated that the inhibition of Aurora B through the use of butein, that has showed anti-tumour activities in different cancers, induced arrest of the cell cycle in G2/M phase and apoptosis in a HCC preclinical study [158].

Notably, pH3S10 was found in 70.6% of HCCs and, together with pH3S28, was up-regulated in aflatoxin B1 (AFB1)-transformed hepatocytes, highlighting a key role of persistent pH3S10 or pH3S28 in chemical carcinogenesis. In fact, the down-regulation of pH3S10 was able to confer resistance to AFB1-induced cell transformation through regulating the transcription of DNA damage response genes [159].

Finally, phosphorylation plays a key role also in regulating other histones. As an example, Li et al. [160] described a novel axis in HCC composed by the Metastasis-associated 1 (MTA1) protein, functioning as an oncogene and promoting HCC progression by regulating the DNA protein kinase proteasomal degradation, and consequent inhibition of phosphorylation of the histone cluster 1 H1 family member c (H1.2) at T146 [160].

##### Others histone post-transcriptional modifications

Protein glycosylation with O-linked *N*-acetylglucosamine (O-GlcNAc) is a PTM highly sensitive to changes in the cellular environment that interacts with other PTMs, playing an essential role. O-GlcNAc is a reversible PTM of serine and threonine residues through a process controlled by O-GlcNActransferases (OGTs) and O-GlcNAcases (OGAs) enzymes, which add or remove the O-GlcNAc to the free hydroxyl group of serine or threonine residues [161].

OGT interacts with various other proteins involved in histone modifications and DNA methylation. Studies have indicated that the O-GlcNAc and OGT are highly expressed in liver cancer, and are associated with HCC progression [162], but literature is lacking about histone glycosylation in HCC.

A study in HCC cells identified the histones H2 and H3 among 110 proteins that showed significant glycosylation, detected through mass spectrometry technology. Histone glycosylation sites were H3K115 and H2BK108, which can also undergo other modifications. However, the interrelationship among glycosylation and other PTMs, and the biological effects of this mark are currently unknown [163].

Histone lysine crotonylation is a relatively new PTM identified by Tan et al. a decade ago [164]. Histone crotonylation, like acetylation, can affect chromatin structure and is enriched on active gene promoters. Its function in cancer is not well known but its levels have been found down-regulated in liver, stomach and kidney carcinomas; and up-regulated in thyroid, oesophagus, colon, pancreas and lung carcinomas. Moreover, in HCC histone lysine crotonylation levels are correlated with metastasis and are increased following HDACs depletion [165]. These results encourage investigations to better understand the biological role of this recently discovered histone modification.

Citrullination is a PTM catalysed by the peptidyl arginine deiminase (PAD) enzyme family that is stimulated by an increased calcium concentration and that can occur in many proteins, such as vimentin, keratin, and some histones like H1, H2A, H3 and H4. PAD prevents histone arginine methylation by directly citrullinating arginine or converting a monomethyl arginine to citrulline, suggesting that histone citrullination antagonizes arginine methylation, thus promoting chromatin decondensation [166].

In hepatitis B virus-associated HCC tissues, a study detected a correlation between the citrullination of histone H3 (H3cit) and Beclin1, an autophagy regulator. The average levels of H3cit and Beclin1 mRNA in HCC were higher than those in non-tumour tissues, and were significantly correlated with vascular invasion and serum AFP levels, suggesting that H3cit could be related to the increase of Beclin1 expression [167]. Another very recent study showed that in HCC histone citrullination by PADI4 may also stabilize the binding of hypoxia-inducible factors to their response elements by increasing access to DNA or by altering interactions with histones [168].

Finally, SUMOylation is a PTM that involves the covalent, but reversible addition of a small ubiquitin-like modifier (SUMO) group to proteins. Due to its capacity to regulate protein activity, this modification has been shown to have profound effects on carcinogenesis and multidrug resistance in HCC [169]. SUMO-specific proteases (SENPs, from 1 to 7) process SUMO precursors to mature forms and also remove SUMO from its targets [170]. In the human genome three functional SUMO isoforms (SUMO-1, SUMO-2, and SUMO-3) are encoded [171]. SUMO-1 was found to be highly expressed in HCC cell lines and HCC specimens respect to non-neoplastic liver tissues [172], and SUMO-2 expression is strongly correlated with patient survival rate [169].

The cellular targets of SUMO proteins favouring HCC progression are multifaceted. SUMO-1 can modify the activity of methyltransferase-like 3, promoting HCC progression via regulating Snail (an epithelial to mesenchymal transition effector) mRNA homeostasis [173]. SUMO-2 regulates liver kinase B1 (LKB1) and exportin-5 (XPO-5) oncogenic activity in HCC [174, 175]. Moreover, SUMOylation appears to play a key role in HCC multidrug resistance. Quin et al. [176] observed that SUMO-1 was significantly increased in 20 HCC compared with matched adjacent non-neoplastic controls. Moreover, the authors demonstrated that multidrug-resistant HCC cell lines showed a markedly high level of SUMO-1-conjugated proteins, associated with an up regulation of SENP1. High level of SENP1 involved HGF/c-Met signals [177]. Silencing of SENP1 in HCC cells reduces the HGF-induced proliferation, invasion and migration, sustaining cell apoptosis and growth arrest. Besides, protein depletion altered the epithelial-to-mesenchymal transition process, with increase of E-cadherin expression, decrease of fibronectin and N-cadherin expression. Moreover, SENP1 promotes hypoxia-induced cancer stemness by HIF-1α deSUMOylation [178, 179]. Recently, SENP5 has been recognized as crucial factor in promoting tumorigenesis process in HCC via regulating DNA damage response [180]. The mRNA and protein levels of SENP5 were over-expressed in 10 pairs of HCC samples and demonstrated that SENP5 was required for the proliferation of HCC cells and tumour growth in vivo.

#### Histone variants

Histone variants are non-canonical (non-allelic) variants of histones, presenting one or a few amino acid differences – with specific expression, localization and species-distribution patterns, that affect chromatin remodelling and histone PTMs [181]. Histone H2A is the canonical histone displaying the highest number of variants: H2A.X, H2A.Z.1, H2A.J, H2A.Z.2.1, H2A.Z.2.2, H2A.Bbd, macroH2A1.1, macroH2A1.2 and macroH2A2 [182]. Hereby, we will summarize selected literature on H2A.Z, macroH2A1 and H2A.X, and HCC.

H2A.Z is one of the histones H2A variants that has 60% similarity with canonical histone H2A in mammalian cells, existing in vertebrates as two distinct genes: H2A.Z.1, encoded by H2AFZ, and H2A.Z.2 subtypes, encoded by H2AFV [183]. Interestingly, in vitro studies demonstrated a potential oncogenic role for H2A.Z isoforms in hepatocarcinogenesis [184–186].

In a large cohort of HCC patients and in HCC cell lines (Hep3B, HepG2, Huh7, PLC/PRF/5, SK-Hep1, SNU182, SNU-368, SNU-449 and SNU-475), H2A.Z.1 was significantly up-regulated, and this correlated with poor prognosis. H2A.Z.1 knockdown suppressed HCC cell growth by transcriptional deregulation of cell cycle proteins, and reduced the metastatic potential of HCC cells by selectively regulating cell cycle components. H2A.Z.1 also promoted proliferation by selectively modulating tumour microenvironment regulatory proteins such as E-cadherin and fibronectin that were elevated [184].

Histone variant macroH2A1 contains a domain that shows 66% homology with histone H2A, which is conserved across various functionally unrelated proteins throughout the animal kingdom, vertebrates and some invertebrates [182]. This variant stands out because of its unique structure, whereby a C-terminal linker connects the histone fold domain to a macrodomain. This macro domain protrudes from the compact nucleosome structure and likely affects the function and organization of the surrounding chromatin. Once in the nucleosome, macroH2A1 may contribute to different cellular processes, including cell cycle regulation, stem cell differentiation, and DNA repair and transcription in somatic and cancer cells [187–191]. MacroH2A1 exists as two alternatively exon-spliced isoforms, macroH2A1.1 and macroH2A1.2. There is also an unrelated variant, called macroH2A2, which is encoded by an independent gene. The protein levels of macroH2A1 splice variants were significantly up regulated in the livers of rodent and human HCC [192]. In particular, in response to the treatment with chemotherapeutic and DNA-demethylating agent 5-aza-deoxycytidine, macroH2A1 expression prevented the acquisition of senescent-like phenotype in HCC cell lines, through induction of global DNA hypomethylation. On the other hand, it has been shown that macroH2A1 knocked down cell lines acquire cancer stem cells features: enhancement of the tumorigenic potential, slow proliferation, resistance to chemotherapy treatments, increasing in mRNA expression of reprogramming genes, and increased glycolysis, reflecting the same phenotype of human undifferentiated and aggressive HCCs, which express a low level of macroH2A1 [193]. Moreover, macroH2A1 was able to rewires carbohydrate and lipid metabolism of HCC cells towards cancer stem cells with increased lipid accumulation through activation of the LXR pathway [194]. Finally, macroH2A1 depleted HCC cells display specific lipidomic and paracrine signatures, which may allow them to escape recognition by the adaptive immune system [192, 194]. Collectively these findings highlighted the critical role of macroH2A1 in HCC diagnosis and prognosis [188, 192, 195].

Phosphorylation of the Ser139 residue of the histone variant H2A.X, forming γ-H2A.X, is an early cellular response to the induction of DNA double-strand breaks. Detection of this phosphorylation event has emerged as a highly specific and sensitive molecular marker for monitoring DNA damage initiation and resolution. Several authors detected an increase of phosphorylated histone variant γ-H2A.X in HCC tissue, highlighting the possibility of use γ-H2A.X also as potential indicator for HCC surveillance [196, 197]. By monitoring the expression levels of γ-H2A.X in HCC tissue samples, obtained from 58 patients (7 cases with hepatitis B virus-positive, 35 with hepatitis C virus-positive, and 16 with unknown aetiology) that underwent hepatic resection, the authors discovered a major enrichment of γ-H2A.X in dysplastic nodules and in non-tumours tissues of HCC as compared with liver cirrhosis without HCC. This accumulation in the pre-neoplastic lesions of HCC supported the idea of an inverse correlation between histological grades of HCC and the level of γ-H2A.X that might play a critical role especially during the early stages of carcinogenesis. It seems that γ-H2A.X could act in promoting HCC angiogenesis via modulation of γ-H2A.X/EGFR/HIF1α/VEGF signalling [198], suggesting a possible combination of γ-H2A.X/EGFR/HIF-1α as a novel marker in the prognosis of HCC and as potential therapeutic target.

### Perspective on application in therapy

At variance with genetic mutations, epigenetic mechanisms such as DNA and histones PTMs, are highly flexible and dynamic. As discussed above, these processes involve reversible enzymatic reactions and specific protein-protein interactions, which make them susceptible to pharmacological manipulation. Indeed, an increasing number of small molecules are being developed to inhibit the deposition of chromatin marks, the elimination of these marks and the interactions of chromatin modifiers and transcriptional regulators with these marks. These new families of compounds are collectively named “epidrugs”, and are showing a great potential for cancer treatment [199, 200]. Interestingly, numerous compounds originally designed for other indications, and that failed to prosper towards their original clinical applications, are being repurposed as promising epidrugs for cancer therapy [201]. Inhibitors of DNMTs and HDACs were among the first epidrugs approved by the Food and Drug Administration for the treatment of haematological malignancies [202, 203]. The anti-tumour activity of these compounds may take place at different levels, including the reprogramming of cancer stemness, the inhibition of tumour initiation and progression, the restoration of cancer cells sensitivity to targeted and conventional chemotherapeutics, and the interaction with the tumour microenvironment [199, 204–207].

#### DNA methylation inhibitors

**Fig. 3:**
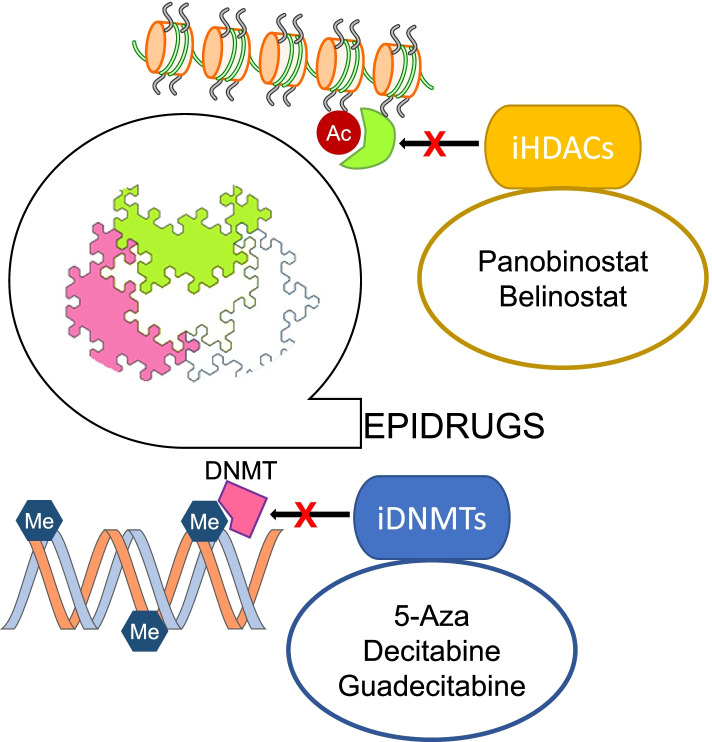
Epigenetic drugs in HCC. Schematic representation of the mechanisms of action of epigenetic drugs tested for HCC therapy

The effects of DNMT inhibitors in HCC in vitro and in vivo models have been studied (Fig. 3). Treatment with first-generation DNMT inhibitors such as 5-azacytidine (5-aza) and 5-aza-2′-deoxycytidine (decitabine) induced the expression of tumour suppressor genes (TSGs), partially recovered HCC cells differentiation, reduced tumorigenicity and enhanced the sensitivity to sorafenib [208, 209]. More advanced DNMT inhibitors with improved pharmacokinetic properties such as guadecitabine (SGI-110) have been developed. Guadecitabine was also shown to induce TSG expression, and to down-regulate the expression of pro-tumorigenic genes through the demethylation of gene body regions [210]. Guadecitabine was clinically tested as a single agent on patients with advanced HCC who failed prior treatment with sorafenib. No disease control was observed, and dose had to be reduced due to toxicity (Table 6). Besides increasing the efficacy of chemotherapy, DNMT inhibitors (DNMTi) may also leverage the efficacy of immunotherapy [211]. This has been experimentally shown for guadecitabine, and ongoing clinical trials are evaluating its effects in combination with immune checkpoints inhibitors (ICI) such as durvalumab in digestive tumours including HCC (Table 6) [212].

**Table 6:** Epigenetics drugs in trial for HCC

DRUGS	Phase	Reference
**DNMTi**		
Guadecitabine (SGI-110) after sorafenib	Phase II	NCT01752933
Guadecitabine (SGI-110) + durvalumab	Phase Ib	NCT03257761
**HDACi**		
Belinostat (PDX-101)	Phase I/II	NCT00321594
Tefinostat	Phase I/II	NCT02759601
Vorinostat + FOLFIRI	Phase I	NCT00537121
Panobinostat + sorafenib	Phase I	NCT00823290
Panobinostat + sorafenib	Phase I	NCT00873002
Resminostat + sorafenib	Phase II	NCT00943449
Resminostat + sorafenib	Phase I/II	NCT02400788
Vorinostat + sorafenib	Phase I	NCT01075113

#### HDAC inhibitors

The efficacy of HDAC inhibitors (HDACi) has also been tested in experimental HCC (Fig. 3). Non-selective HDACi like panobinostat reduced HCC cells proliferation, reprogrammed cancer cells metabolism and reduced tumour angiogenesis [213]. Another pan-HDACi, belinostat, also demonstrated preclinical efficacy, and some activity when tested as second line treatment in HCC patients with unresectable tumours (Table 6) [214]. Interestingly, combination of belinostat with ICI resulted in enhanced efficacy of immunotherapy in an experimental model of HCC [215]. Tefinostat, another HDACi, has been also tested as single agent in HCC patients in a dose escalation trial but no results are still available (Table 6). Vorinostat, in combination with irinotecan, fluorouracil and leucovorin (FOLFIRI), was also evaluated in patients with digestive cancers and HCC (Table 6). The HDACi panobinostat has been clinically evaluated in combination with sorafenib in a limited number of patients with advanced HCC, but no results are available (Table 6). The combination of the HDACi resminostat with sorafenib was evaluated as second-line therapy in patients with advanced HCC. This combination was safe and showed early signs of efficacy [216]. A subsequent Asian study compared first-line therapy with resminostat plus sorafenib versus sorafenib monotherapy [217]. This approach did not achieve any improvement over sorafenib monotherapy, however some benefit was appreciated in a subgroup of patients including those with hepatitis B virus-related HCC (Table 6). The combination of vorinostat with sorafenib has also been evaluated. Although some patients had durable disease control, the inclusion of vorinostat led to toxicity in a majority of patients [218] (Table 6).

#### Other epigenetic drugs

The development of other epidrugs, such as inhibitors of histones methyltransferases, as cancer therapies is also actively pursued [219]. For instance, GSK126, an EZH2 inhibitor already used in trial against different types of lymphomas, has been shown to enhance natural killer cell-mediated HCC cell death [220]. Targeting of other histone methyltransferases like G9a (EHMT2) has also shown preclinical efficacy in HCC models [221]. As can be inferred from all the information summarized along this review, epigenetic mechanisms are quite complex and very often work in concert in the normal and also pathological regulation of gene expression. These notions have led to the development of new types of molecules combining dual inhibitory activities acting on different epigenetic targets. In the context of hepatocarcinogenesis, one recent example of this new family of compounds is CM272, a dual inhibitor of DNMT1 and G9a, enzymes that co-ordinately mediates the silencing of TSG and promote cancer growth [222]. Combined inhibition of DNMT1 and G9a by CM272 showed anti-tumour activity in experimental HCC, and also has a strong effect on the fibrogenic reaction, which is closely linked to HCC development and progression [223, 224]. Pharmacological targeting of epigenetic readers is also attracting much attention [225]. The expression of the acetylated H3 and H4 reader BRD4 is enhanced in HCC [225, 226], and treatment with the BRD4 inhibitor JQ-1 reduced HCC cell growth and survival [227, 228]. Importantly, targeting BRD4 also enhanced ICI efficacy in experimental HCC [229]. Taken together, these findings support the potential of epigenetic strategies for the treatment of HCC. Nevertheless, the efficacy and safety of these approaches cannot be properly established until our knowledge of the fundamental intricacies of epigenetic regulation is not further advanced. The evaluation of safer and more efficacious combination regimens with conventional therapies, and ICI-based therapies, need also to be carefully addressed.

## Conclusion

As described in the present review, epigenetic marks can be either indicators of the presence of disease and/or specific biomarkers for disease stage and prognosis. Also, technical approaches and results from epigenetic studies performed in adult HCC may be used to explore the most common aberrant molecular pathways, to stratify tumour subtypes, and to guide research efforts in paediatric liver cancer as well, as recently reported [230–232].

Most preclinical studies demonstrated that epigenetic remodelling is potentially reversible by drugs. The list of available epigenetic modifiers and inhibitors is progressively growing. Therefore, in the era of precision medicine the unravelling of the epigenetic modifications occurring in HCC may provide new tips for the screening of potential therapeutic targets and the study of personalized interventions strategies for the management of this tumour. When considering combination therapies with conventional chemotherapeutics, other targeted agents, or ICIs, the optimization of scheduling strategies is a very relevant issue. In order to maximize efficacy, potentiate synergisms and overcome resistances, the simultaneous, sequential or alternate administration of epigenetic drugs needs to be taken into account [233]. As mentioned above, toxicity has been observed in the few clinical trials performed on HCC patients. Therefore, adverse events and dosage must be carefully monitored, particularly in patients with an underlying liver disease like most HCC patients. Finally, the identification of predictive biomarkers of response is also a challenge, particularly when mutations in epigenetic targets are not frequent in HCC. Further molecular analyses of samples from patients enrolled in clinical trials may facilitate biomarker discovery.

## Data Availability

Not applicable.

## Abbreviations

HCC: Hepatocellular carcinoma
HBV: hepatitis B virus
HCV: hepatitis C virus
NASH: non-alcoholic steatohepatitis
NAFLD: non-alcoholic fatty liver disease
AFP: α-fetoprotein
ncRNAs: Non-coding RNAs
sncRNAs: small/short noncoding RNAs
miRNAs: microRNAs
piRNAs: piwi-interacting RNAs
endo-siRNAs: endogenous short interfering RNAs
snoRNAs: small nucleolar RNA
lncRNAs: long noncoding RNA
circRNA: circular RNA
3’UTR: 3′-untranslated region
ceRNA: competing endogenous RNA
PTMs: post-translational modifications
DNMTs: DNA methyltransferases
C: Cytosine base
G: Guanine base
CpGIs: CpG islands
H3K4me: histone 3 lysine 4 methylation
H3K9me: histone 3 lysine 9 methylation
H3K27me: histone 3 lysine 27 methylation
PRC1: polycomb repressive complex 1
PRC2: polycomb repressive complex 2
H2AK118ub: histone H2A lysine 118 monoubiquitination
H2AK119ub: histone H2A lysine 119 monoubiquitination
EZH1/2: enhancer of zeste homolog 1 and 2
SUZ12: suppressor of zeste 12
EED: embryonic ectoderm development
RBBP4/7: retinoblastoma-binding protein 4/7
FAK: focal adhesion kinase
H4K20me3: histone H4 lysine 20 trimethylation
H4K16: histone H4 lysine 16
HATs: histone acetyltransferases
HDACs: histone deacetylases
H3K9ac: H3K9 acetylation
HMGA2: High-mobility group protein A2
HOX: homeobox
USP22: Ubiquitin-specific protease 22
pH3S10: histone H3 serine 10 phosphorylation
pH3S28: histone H3 serine 28 phosphorylation
AFB1: aflatoxin B1
MTA1: Metastasis-associated 1
H1.2: histone cluster 1 H1 family member c
O-GlcNAc: O-linked N-acetylglucosamine
OGTs: O-GlcNActransferases
OGAs: O-GlcNAcases
PAD: peptidyl arginine deiminase
H3cit: citrullination of histone H3
SUMO: small ubiquitin-like modifier
SENPs: SUMO-specific proteases
LKB1: liver kinase B1
XPO-5: exportin-5
5-aza: 5-azacytidine
TSGs: Tumour suppressor genes
ICI: Immune checkpoints inhibitors
HDACi: HDAC inhibitor
